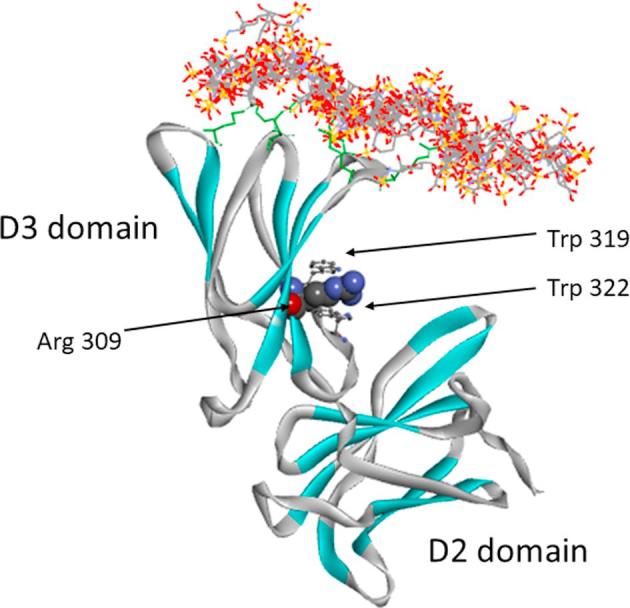# The localisation of the heparin binding sites of human and murine interleukin-12 within the carboxyterminal domain of the P40 subunit

**DOI:** 10.1016/j.cyto.2018.04.014

**Published:** 2018-10

**Authors:** Pascale Garnier, Rosemary Mummery, Mark J. Forster, Barbara Mulloy, Roslyn V. Gibbs, Christopher C. Rider

**Affiliations:** a Centre for Biomedical Sciences, School of Biological Sciences, Royal Holloway University of London, Egham Hill, Egham, Surrey TW20 0EX, UK; b National Institute for Biological Standards and Control, Blanche Lane, South Mimms, Potters Bar, Herts EN6 3QG, UK; c Scientific Computing Department, Daresbury Laboratory, Warrington, Cheshire WA4 4AD, UK^2^; d School of Pharmacy and Biomedical Sciences, University of Portsmouth, White Swan Road, Portsmouth, Hants PO1 2DT, UK

**Keywords:** Interleukin-12, Interleukin-23, Heparin, Heparan sulfate, P40 subunit, Docking calculations, BMP, bone morphogenetic protein, CS, chondroitin sulphate, FGF, fibroblast growth factor, FGFR, fibroblast growth factor receptor, GAG, glycosaminoglycan, GDNF, glial cell line-derived neurotrophic factor, γ-IFN, γ-interferon, HS, heparan sulfate, h, human, IL, interleukin, m, murine, NK, natural killer, r, recombinant, TNF, tumour necrosis factor

## Abstract

We have previously shown that the heterodimeric cytokine interleukin-12, and the homodimer of its larger subunit p40, both bind to heparin and heparan sulfate with relatively high affinity. In the present study we characterised these interactions using a series of chemically modified heparins as competitive inhibitors. Human interleukin-12 and p40 homodimer show indistinguishable binding profiles with a panel of heparin derivatives, but that of murine interleukin-12 is distinct. Heparin markedly protects the human and murine p40 subunits, but not the p35 subunits, from cleavage by the bacterial endoprotease LysC, further implicating the larger subunit as the location of the heparin binding site. Moreover the essential role of the carboxyterminal D3 domain in heparin binding is established by the failure of a truncated construct of the p40 subunit lacking this domain to bind. Predictive docking calculations indicate that a cluster of basic residues at the tip of the exposed C′D′ loop within D3 is important in heparin binding. However since the human and murine C′D′ loops differ considerably in length, the mode and three dimensional orientation of heparin binding are likely to differ substantially between the human and murine p40s. Thus overall the binding of IL-12 via its p40 subunit to heparin-related polysaccharides of the extracellular matrix appears to be functionally important since it has been conserved across mammalian species despite this structural divergence.

## Highlights

• We demonstrate differences in the specificity of heparin binding between human and murine IL-12s.
• Heparin predominantly protects the p40 subunit against proteolysis by LysC.
• A truncated IL-12 polypeptide lacking the carboxyterminal D3 domain fails to bind heparin.
• The C′D′ loop of the D3 domain contains a large cluster of surface accessible basic residues.
• This loop is the region of greatest sequence variation between murine and human p40s.

## Graphical abstract

**Figure:**
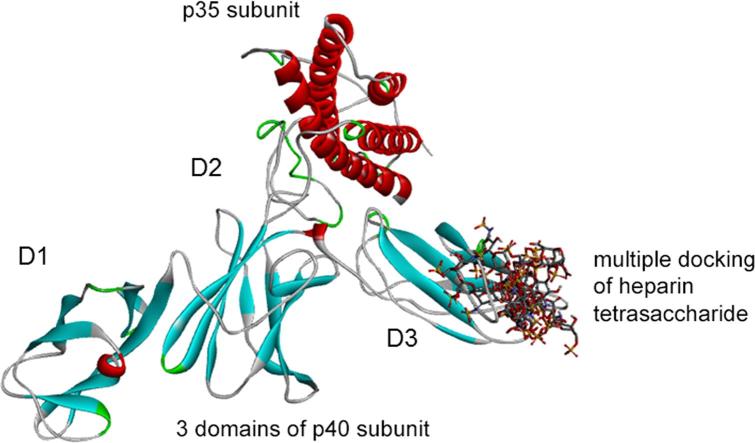

## 1 Introduction

Interleukin 12 (IL-12) plays a key role in establishing cell-mediated, T_H_1-type, immune responses by stimulating NK and T lymphocytes to secrete γ-IFN (for reviews see [1], [2]). The major cellular sources of IL-12 are activated macrophages, monocytes and neutrophils. In turn, γ-IFN stimulates further production of IL-12, thus giving rise to a positive feedback loop at the initiation of T_H_1 responses. IL-12 is a relatively large cytokine, M_r_ 70kD, being a heterodimer of two disulphide-bridged subunits, p40 and p35. The p40 subunit also occurs as a component of IL-23, in which the smaller p35 subunit is replaced by a structurally related subunit, p19 [2]. IL-23 is a pro-inflammatory and immunostimulatory cytokine expressed by antigen presenting cells upon activation. However IL-23 stimulates the T_H_17 lymphocyte subset, thereby promoting both early responses to microbial infection and chronic autoimmune states. The p35 subunit is also found in IL-35, an immunosuppressive cytokine [3]. The high affinity cell surface signalling receptor for IL-12 is a heterodimer of the IL-12Rβ1 polypeptide, which is also a component of the IL-23 receptor, and IL-12Rβ2, which is shared with the IL-35 receptor [3]. Free p40 is also found in serum in a number of disease states and in efforts to identify the biological role of this polypeptide a number of binding proteins have been identified, including the CD5 glycoprotein [4].

The pivotal role of IL-12 in initiating and promoting T_H_1 immune responses was convincingly demonstrated in IL-12 p40 deficient mice, which show greatly impaired γ-IFN responses to antigenic challenge [5], and are unable to mount an effective T_H_1 response to *Mycobacterium tuberculosis* infection [6]. Moreover both p40^−/−^ and p35^−/−^ mice, despite having an otherwise resistant genetic background, show susceptibility to *Leishmania major* infection and polarised T_H_2 immune responses [7]. In humans, deficiencies in IL-12 production and signalling have been found to cause susceptibility to disseminated *Salmonella* and mycobacterial infections, including tuberculosis [8], [9]. Based on previous evidence from experimental rodent models of disease, IL-12 and p40 have become targets of interest in a number of chronic human diseases with autoimmune and inflammatory aspects, including psoriasis, psoriatic arthritis, Crohn’s disease, ulcerative colitis, rheumatoid arthritis and multiple sclerosis [reviewed in 10]. Ustekinumab, a humanised p40-specific monoclonal antibody which blocks IL-12 and IL-23 signalling [11], is now an approved therapeutic agent for psoriasis and psoriatic arthritis [12], [13], and also for the treatment of moderate to severe Crohn’s disease in patients refractory to anti-TNF treatment [14]. In addition IL-12 has long been recognised to have anti-tumour activities [15], and the localised delivery of IL-12 remains under investigation in the experimental immunotherapy of cancer [16], [17], [18].

The p35 subunit of IL-12 shares weak sequence homology with IL-6, a 4 α-helix bundle cytokine [19]. Likewise an extensive if weak sequence homology has been noted between p40 and the IL-6 receptor polypeptide [20]. IL-12 can therefore be conceptualised as a 4-α helix bundle cytokine covalently pre-bound to a soluble class I cytokine receptor chain. The high resolution crystallographic structures of human IL-12 accord entirely with this paradigm [21], [22]. The p35 subunit indeed has a 4 α-helix conformation, whereas p40 has 3 distinct fibronectin-like Ig domains. The p35 subunit docks into a hinge region between the central p40 domain, D2, and the carboxyterminal p40 domain, D3. The epitope for ustekinumab lies within the aminoterminal domain of p40, D1 [22]. Recent structural studies of IL-23 show that briakinumab, another IL-12 and IL-23 blocking monoclonal antibody, binds to p40 at the same site [23].

We have previously reported that human and murine IL-12 bind with comparatively high affinity to the sulfated glycosaminoglycans (GAGs) heparin and heparan sulfate, HS [24]. This binding appears to involve highly sulfated motifs within the GAG chains. Since several 4 α-helix cytokines including ILs-2 [25], -4 [26], -5 [27], -6 [28], and -7 [29], are heparin-binding cytokines, it might be surmised that IL-12 binds to sulfated GAGs via p35. However our finding that the human p40 dimer binds to heparin and HS in a manner indistinguishable from human IL-12 indicates instead that the binding site resides within p40 [24].

The binding of a cytokine to heparin-related polysaccharides of the extracellular matrix and cell surface will serve to retain the cytokine close to its site of secretion within tissue microcompartments [30]. Given the biological role of IL-12 as an activating signal for T and NK lymphocytes secreted by antigen presenting cells, the localised delivery of IL-12 is likely to be physiologically significant in the context of the immunological synapse which forms as a tight physical contact between antigen presenting cells and T cells. Interestingly γ-IFN, which is produced in response to IL-12, is also a heparin/HS binding cytokine [31], suggesting that these GAGs are important in localising the initiation of T_H_1 immune responses.

As has been well studied in the case of FGFs, heparin/HS may also function as cytokine co-receptors, by participating in the formation of cytokine-receptor complexes and thereby promoting signalling activity [reviewed in 32]. Whether sulphated GAGs function as co-receptors for IL-12 remains unclear, although we have shown that stripping the murine natural killer cell line KY-1 of the GAG chondroitin sulfate B, greatly reduces responsiveness to IL-12 in terms of interferon-γ secretion, and that chondroitin sulfate B competes for the heparin binding site on IL-12, albeit with lower affinity [33].

Here we investigate further the structural basis of the interaction between IL-12 and sulphated GAGs. We report experimental findings which clearly implicate the carboxyterminal domain, D3, of the p40 subunit as the site of heparin binding. We have also employed theoretical docking calculations which predict that key amino acid residues involved in heparin binding are located in basically charged clusters on flexible, exposed polypeptide loops near the carboxyterminus of p40 D3, a region of appreciable structural divergence between murine and human IL-12.

## 2 Materials and methods

### 2.1 Materials

Recombinant murine and human IL-12s (rmIL-12 and rhIL-12), rhIL-12 p40, and goat polyclonal anti-rmIL-12 and anti-rhIL-12 were purchased from R&D Systems Europe Ltd. (Abingdon, Oxon, UK). Alkaline phosphatase- and horseradish peroxidase-coupled anti-goat Ig, endoproteinase LysC (EC 3.4.21.50) and *p*-nitrophenyl phosphate tablets were all obtained from the Sigma Chemical Co., St. Louis, MO, USA. A series of chemically modified heparins, were prepared and structurally characterised as fully described elsewhere [34]. PCR primers were obtained from MWG Biotech (Edersberg, Germany). Murine IL-12 p35 and p40 cDNA clones in pBluescript were obtained from the American Type Culture Collection (ATCC, Manassas, VA, USA). Restriction endonucleases were purchased from Promega Corporation (Madison, WI, USA).

### 2.2 Heparin binding ELISA, proteolytic digestion and Western blotting

The binding of rIL-12 and its derivatives to an immobilised heparin-BSA complex was studied by ELISA as described previously [24], [25]. For digestion with endoproteinase LysC, rIL-12 and p40 (1.25 μg/ml) were incubated with 2 μg/ml enzyme in 20 mM Tris–HCl buffer, pH8.0, at 37 °C. Aliquots, 20 μl, were removed at different digestion times and immediately boiled in SDS sample buffer. Digested samples were separated by SDS PAGE on 15% (wt/vol) polyacrylamide gels and electroblotted on to nitrocellulose. All other samples for Western blotting were separated on 12% (wt/vol) gels. After blocking in PBS containing 5% (wt/vol) fat-depleted dried skimmed milk powder and 0.05% (vol/vol) Tween for 30 min, followed by washing in PBS, the blot was incubated overnight at 4 °C in blocking buffer containing goat anti-IL-12 antibody at 1/2000 dilution. After washing in blocking buffer, the blot was incubated with peroxidase-coupled anti-goat Ig diluted 1/2000 for 45 min. Finally IL-12 immunoreactivity was detected by chemiluminescence reagents (SuperSignal West Pico, Perbio, Tattenhall, Cheshire, UK).

### 2.3 Murine IL-12 gene constructs and site directed mutagenesis

Murine p35 and p40 cDNAs were cloned directly into the pTriEx vector (Novagen, Darmstadt, Germany) for expression in mammalian cells. Following the approach of Lieschke et al. [35], a single chain construct, psc70, was generated by splicing the sequence for p40 at its carboxyterminus via a flexible linker, (Gly_4_Ser)_3_*,* to codon 23 of the p35 cDNA, which encodes Arg, the first residue following the presumptive signal sequence. Within the p40 cDNA, an *Nco1* restriction site was introduced at the aminoterminal end by a single base change in the second codon (TGT to GGT), and the stop codon was deleted at the 3′ extremity. These changes were introduced by PCR amplification using the following primers: p40-*Nco1* (sense), 5′-CAAAGCACCTAGGGTCCTCAGAAG-3′; p40-*Nco1* (antisense), 5′-TTTAAAGGATCCACCACCGCCCGAGCCACCGCCACCGGATCGGACCCT-3′; p35-linker (sense), 5′-AAATTTGGATCCGGTGGCGCCGGATCTAGGGTCATTCCAGTCTCTGGA-3′; p35-C terminus (antisense) 5′-AAAAGCTGGAGCTCCACCGCG-3′, with the *Nco1* restriction sites shown underlined. P40 and p35 PCR products were respectively digested with *Nco1/BamH1* and *BamH1/Not1* enzymes, purified electrophoretically, ligated and subcloned into the pGEM-T Easy vector (Promega, Southampton, Hants., UK).

In order to produce the p40 variant, p40ΔD3*,* which lacks the D3 domain, a stop codon was introduced at the end of the D2 codon by PCR using the QuickChange site-directed mutagenesis kit (Stratagene Europe, Amsterdam, Netherlands). The mutagenesis primers used for this were; sense, 5′-CTTCATCAGGGACATCATCTAACCAGACCCGCCCAAGAAC-3′ , and, antisense, 5′ – GTTCTTGGGCGGGTCTGGTTAGATGATGTCCCTGATGAAG-3′, with the nucleotide substitution underlined. Parental strands were then digested with *Dpn1* and PCR products were transformed into XL-1B (Stratagene). All coding sequence constructs were confirmed by commercial DNA sequencing (MWG Biotech, Milton Keynes, UK) using universal and internal primers.

### 2.4 Expression of recombinant IL-12 constructs

African green monkey kidney COS-7 cells were grown in DMEM supplemented with 10% (vol/vol) FCS, 2 mM glutamine and 100 μg/ml gentamycin. Cells were transfected at 70–80% confluence with 2 μg plasmid DNA using GeneJuice transfection reagent (Novagen, Nottingham, UK) following the supplier’s instructions. 18hrs after transfection, the culture medium was replaced with a serum-free variant. After a further 18hrs, conditioned medium, or cells extracted in PBS containing 1% (vol/vol) NP40 and protease inhibitor set (Roche Applied Science, Lewes, East Sussex, UK), were immediately denatured by boiling in SDS sample buffer containing 5% (vol/vol) 2-mercaptoethanol analysed by Western blotting as described above. The IL-12 bioactivity of conditioned supernatants from transfected cell cultures was assayed according to their ability to induce γ-IFN expression in the murine KY-1 natural killer cell line [36] as described fully elsewhere [33].

### 2.5 Molecular modelling of human and murine IL-12 and predictive docking with heparin oligosaccharides

Visualisation of the molecular models used the programs Insight and Discovery Studio Visualiser (Accelrys, Cambridge, UK). Molecular surfaces coloured by interpolated charge were generated in Discovery Studio Visualiser. Complete sequences of human and murine IL-12, and their constituent sub-units, were modelled on the basis of crystal structures; a description of the homology modelling protocols and strategy is presented in the Supplementary Material. Sequence alignments for the p40 subunits are shown in Fig. S1, together with the extent of the residues resolved within each of the crystal structures.

Docking calculations of heparin model ligands to protein structures 1F42 (human p40) and 1F45 (human p40 and p35 heterodimer) [21] were performed as previously described, using the program Autodock version 2.4 [37], in the virtual space of a cube of side 80 Å. This program allows limited flexibility in the ligand structure, but uses a rigid body protein approximation. As this protocol limits the size of protein model which can be used, subunits of the IL-12 protein models were used for docking calculations, and for both human and mouse models, the p40 subunit was split into D1 (N-terminal), and a two-domain D2-D3 model. A p35-D2-D3 model was also investigated. Two heparin pentasaccharide model ligands consisted of three GlcN2S6S residues separated by IdoA2S residues, with the IdoA2S residues in the ^1^C_4_ and ^2^S_0_ ring forms respectively; an endecasaccharide model consisted of six GlcN2S6S separated by IdoA2S residues. These model ligands have fixed glycosidic torsion angles taken from the PDB file 1HPN, a solution NMR structure [38]. Flexibility was allowed for all exocyclic torsion angles in the pentasaccharide models, but was not allowed for the endecasaccharide model. Partial atomic charges were obtained by *ab initio* quantum chemistry calculations on 1-OMe 4-OMe substituted monosaccharides, in the Jaguar program (Schrodinger Inc, Portland, Oregon, USA). Intermolecular interaction energies calculated in Autodock for the heparin/IL-12 complexes were used to rank calculated complexes. Docking calculations for IL-12 based on the crystal structure 3HMX, and p40 models based on the structures 4EO8 and 3DUH were also carried out using the ClusPro server v. 2.0, (http://cluspro.bu.edu/) [39], with the tetrasaccharide heparin ligand option offered by the server.

## 3 Results

### 3.1 Specificity of heparin binding

**Fig. 1:**
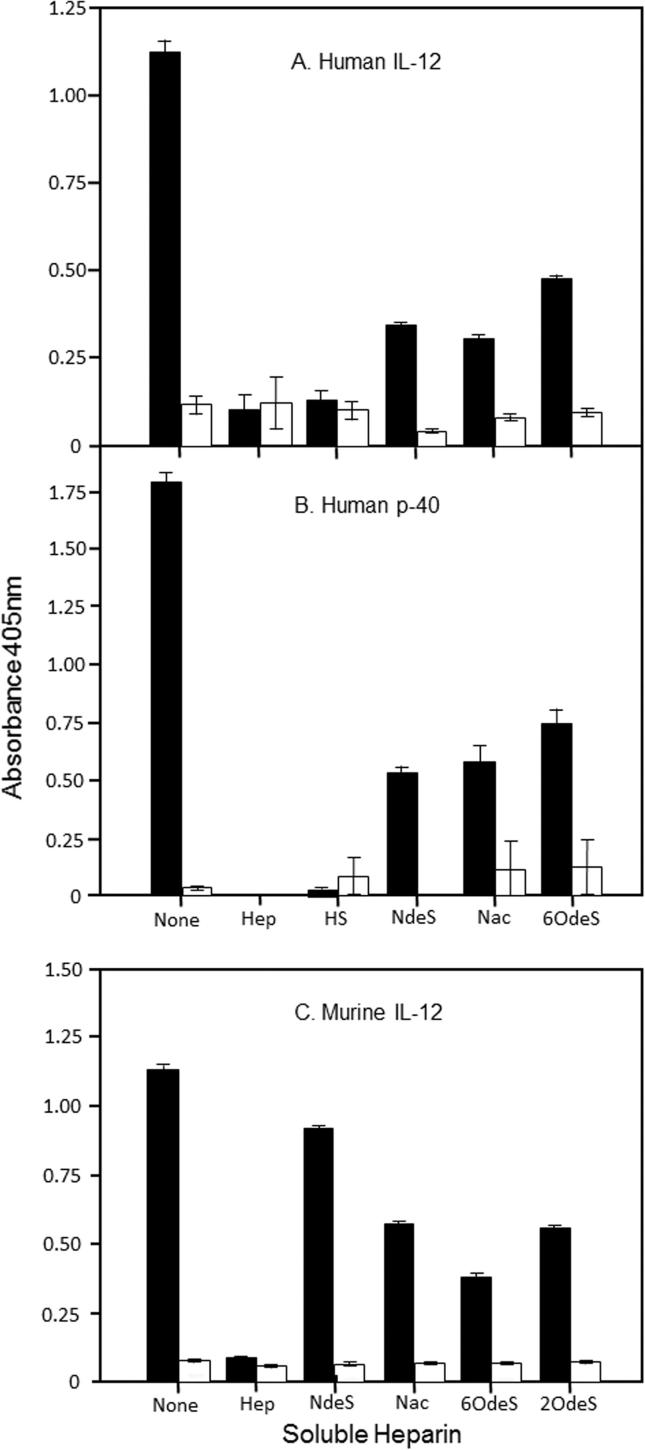
Heparin-related glycosaminoglycans as competitors of the binding of IL-12 to immobilised heparin. Competitive ELISA of the binding to immobilised heparin BSA complex of (A) human recombinant IL-12, (B) human recombinant p40, (C) murine recombinant IL-12. Throughout, 8 ng/well recombinant cytokine was preincubated prior to addition to the ELISA plate wells, in the absence (None) or presence of 50 μg/ml heparin-related glycosaminoglycan as follows: intact bovine lung heparin (Hep), bovine heparan sulfate (HS), *N*-desulfated heparin (NdeS), *N*-sulfated re-*N*-acetylated heparin (Nac), selectively 6-*O*-desulfated heparin (6OdeS), or selectively 2-*O*-desulfated heparin (2OdeS). Solid columns indicate binding to heparin-BSA coated wells, and open columns represent binding to wells coated with mock-conjugated BSA. Each column represents means of triplicate wells, and error bars show ± standard error of the mean.

We sought to characterise the interaction between rIL-12 and heparin/HS using a series of chemically modified heparin preparations in which specific sulfate moieties had been removed. We examined the effects of these preparations on the binding of rhIL-12 to an immobilised synthetic heparin-BSA complex using an ELISA method as previously described [25]. As shown in Fig. 1 panel A, and entirely consistent with our previously published findings [24], rhIL-12 binds strongly to wells coated with this complex, whereas wells coated instead with mock-conjugated BSA show only minimal binding. Preincubation for 15 min of rhIL-12 with soluble heparin and HS reduces binding to the complex to the low background levels which occur in control wells coated with mock-conjugated BSA. Thus this shows complete competition for the specific binding to immobilised heparin. However at the same concentration, 50 μg/ml, soluble chemically modified heparins provide only partial inhibitions. Over a series of three independent experiments, the selectively *N*-desulfated, *N*-desulfated/*N***-**reacetylated, and 6-*O*-desulfated preparations were found to give average inhibitions of 65, 70 and 56% respectively. The same experiment conducted instead with the rhIL-12 p40 homodimer, Fig. 1 panel B, yields essentially indistinguishable results. However, when rmIL-12 was investigated (Fig. 1 panel C), the *N*-desulfated preparation is substantially less potent than the other preparations, including the *N*-desulfated/*N*-acetylated and 6-*O*-sulphated derivatives, giving only some 20% inhibition of binding. This indicates a species difference in the specificity of the IL-12 heparin binding interactions.

### 3.2 Protection from proteolysis

**Fig. 2:**
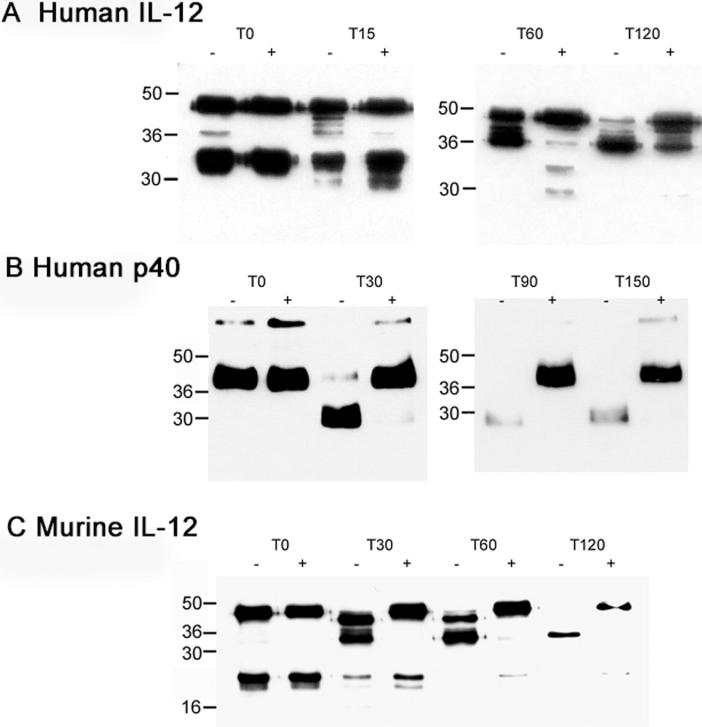
Digestion of IL-12 with Lys-C in the presence and absence of heparin. Human IL-12 (panel A), human p40 (panel B), and murine IL-12 (panel C) were each digested with Lys-C at 37 °C in the presence (+) and absence (−) of 50 μg/ml heparin. The time in minutes of sampling after the start of digestion is shown above each pair of tracks. Each panel is representative of triplicate experiments. Digestion products were separated by SDS PAGE on 15% (wt/vol) polyacrylamide gels and immunoblotted with the appropriate polyclonal antibody. The migration of molecular weight markers are indicated in kD.

We next investigated the effect of heparin on the digestion of the subunits of IL-12 with the endoproteinase Lys-C. This enzyme cleaves polypeptide chains at lysine, which by possessing a basically charged sidechain is, along with arginine, a key residue in the binding sites for the acidic GAGs. The digestion products were examined by immunoblotting. As may be seen in Fig. 2 panel A, the undigested human rhIL-12, T_O_ material, (top left) shows two strong immunoreactive bands of apparent M_r_s 45 and 35kD, corresponding respectively to the p40 and p35 subunits of IL-12. Under the conditions employed, the p35 band proves to be highly susceptible to digestion, as in the absence of heparin the immunoreactive band has completely disappeared by 60 min, and is already considerably diminished by 15 min. The high resolution structures of IL-12 [21], [22] reveal that a number of the lysine residues in p35 are highly exposed within the cytokine structure. The presence of heparin provides only minor protection as seen by reduced degradation at 15 min, however by 60 min degradation appears almost complete.

The human IL-12 45kD band is more resistant to digestion as in the absence of heparin, 45kD immunoreactivity is seen throughout the digestion period, although there is a progressive downward shift of the major intensity to a position between the original p40 and p35 bands. This indicates that a major immunoreactive intermediate on the degradation pathway of p40 is a slightly smaller polypeptide suggesting that sites susceptible to Lys C must be located relatively close to one or both ends of the p40 polypeptide chain. Heparin results in more pronounced protection of the human p40 subunit than that seen with p35. After 120 min digestion in the presence of heparin the major intensity at 45kD still migrates at an unchanged position, in complete contrast to the near total loss of this band seen in the absence of heparin. Although the more rapidly migrating band begins to appear in the presence of heparin at later digestion times, it remains only a minor constituent of the total immunoreactivity.

As shown in Fig. 2 panel B, similar Lys C digestion of the recombinant human p40 homodimer in the absence of heparin, demonstrated that by 30 min virtually all of the p40 immunoreactivity has been shifted to a faster migrating position corresponding to the clipped product discussed above for Fig. 2 panel A. By contrast, in the presence of heparin the intact p40 immunoreactivity remains undiminished throughout the digestion period, and there is no evidence of the lower M_r_ degradation intermediate under the conditions employed.

On the similar digestion of murine rIL-12 (Fig. 2 panel C), in the absence of heparin, within 30 min all the p40 immunoreactivity is faster migrating, with two major bands discernible. One of these shows a slight shift in migration, whereas the second migrates substantially faster. This suggests two stages of clipping relatively near the ends of the polypeptide. By 60 min, the faster band predominates, and by 120 min, it is the only remaining band albeit at very low intensity. As with the human IL-12, heparin exhibits a clear protective effect on murine p40, since in its presence at 60 min, the original p40 band is essentially unchanged in migration or intensity. Moreover in the presence of heparin at no stage do either of the two major p40 degradation bands appear. The murine p35, like its human counterpart, is more rapidly degraded than the larger subunit, and similarly shows only modest protection by heparin. Overall these observations show that for both murine and human IL-12, heparin protects primarily the p40 subunit, at sites adjacent to the termini of the polypeptide.

We conducted in parallel, similar incubations of Lys-C with rhIL-10 in the presence and absence of heparin. In this case heparin failed to protect the cytokine, indeed degradation of IL-10 appeared faster in the presence of heparin (data not shown). These digestions establish that the effects observed with IL-12 are the specific outcomes of interactions of heparin with this particular cytokine.

### 3.3 Recombinant IL-12 constructs

**Fig. 3:**
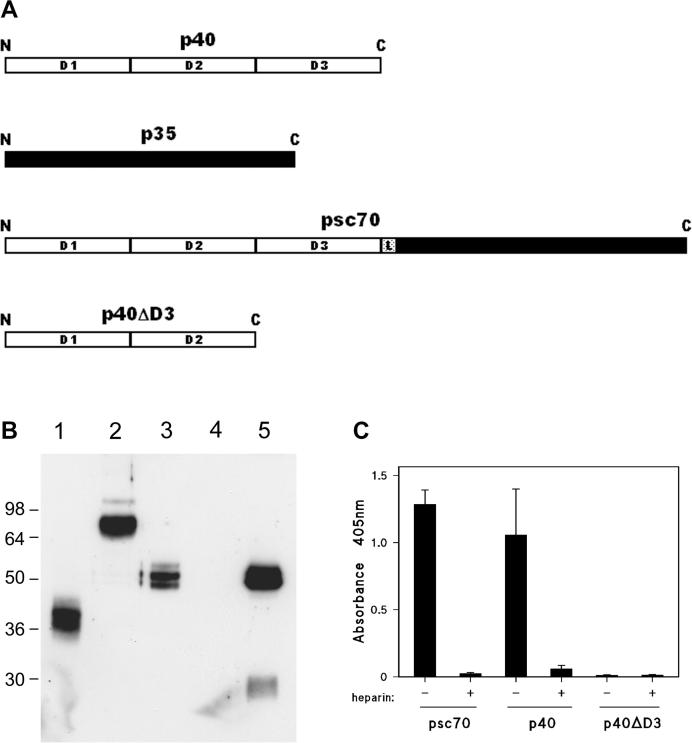
Heparin binding properties of recombinant murine IL-12 constructs. (A) Schematic representation of the expressed constructs. The p40 polypeptide is represented as an open ribbon with the three domains indicated. The p35 polypeptide is represented as a filled ribbon. In the psc70 construct, the flexible linker 15-mer peptide sequence used to fuse the subunit polypeptides is represented by a shaded box marked L. (B) Western blots of conditioned supernatants from transfected COS cells. The individual tracks were loaded with supernatants from cultures transfected with (1) p40ΔD3, (2) psc70, (3) p40, and (4) p35. Track (5) was loaded with commercially expressed recombinant murine IL-12. Track (4) is a maximal loading, whereas tracks (1)–(3) were downloaded at least three fold to provide comparable densities on immunodevelopment with each other and the p40 band of track (5). Samples were separated on 12% polyacrylamide gels. The migration of molecular weight markers are indicated in kD. (C) Heparin-binding ELISA of IL-12 constructs. ELISA plate wells were coated with synthetic heparin-BSA conjugate and used as a capture surface for samples of condition supernatants from transfected COS-7 cells which had been preincubated in the absence (−) or presence (+) of 5 μg/ml soluble heparin. Each column shows the mean of quadruplicate wells and error bars represent the standard error of the mean. The loadings of the supernatants for psc70 and p40 were adjusted as for panel B, except that p40ΔD3 was uploaded 2-fold to maximise any possible signal. The data shown are from a single experiment, representative of a series of three.

In order to locate the heparin binding site within murine IL-12, we expressed recombinant constructs encoding the individual murine IL-12 subunits, and also a single chain variant of IL-12, psc70, in which the subunits are fused using a flexible linker. The latter has previously been shown to possess high *in vivo* bioactivity [35]. We also expressed a truncated variant of p40, p40ΔD3, in which the D3 domain is deleted. These constructs are represented schematically in Fig. 3A. As shown in Fig. 3B, in the conditioned supernatants harvested from transfected cells strong immunoreactive bands were detectable by Western blotting at the predicted M_r_s for psc70 (lane 2) and p40 (lane 3), with the latter resolving as a doublet. However in the case of attempted p35 expression (lane 4), there is no clear band of around 35kD co-migrating with the lower band detected in commercial rmIL-12 (lane 5). This may be in part because the expression and secretion of p35 occurs less efficiently compared to the p40 subunit [40], but also because the p35 subunit is less immunogenic, and therefore less readily detectable by immunological techniques [41]. The weak detection of p35 compared to p40 is indeed seen with the commercial rmIL-12 in lane 5. For the p40ΔD3 construct (lane 1), a strong immunoreactive band migrates with an apparent M_r_ of approximately 35kD, consistent with the predicted size. The conditioned supernatant from COS cells transfected to express psc70 was found to have high IL-12 bioactivity as determined by the induction of γ-IFN secretion in a natural killer cell line [36]. No such bioactivity could be detected in the supernatants of COS cells either mock transfected, or transfected to express the p40 polypeptide (data not shown), findings entirely consistent with previous studies [1], [35].

The conditioned supernatants from the transfected COS cells were analysed by heparin binding ELISA. In these ELISAs, sample volumes were routinely adjusted to provide comparable loadings as judged by band intensities in Western blotting. As may be seen in Fig. 3C, strong heparin-binding IL-12 immunoreactivity was detectable in the conditioned supernatant from psc70 transfected COS cells. This binding immunoreactivity was completely displaced in the presence of a low concentration of soluble heparin. Thus the psc70 immunoreactivity behaved in an identical manner to commercial, purified rmIL-12 as observed elsewhere in this study (Fig. 1C), and previously [24]. The conditioned supernatant from p40-transfected cells provided comparably strong heparin-binding IL-12 immunoreactivity which was also completely displaced by soluble heparin, again consistent with our previous results for commercial purified recombinant human p40 (Fig. 1B and [24]). By contrast the p40ΔD3 supernatant failed to provide heparin-binding IL-12 immunoreactivity, despite the same sample exhibiting strong IL-12 immunoreactivity on Western blotting (Fig. 3B). Indeed, compared to the Western blotting experiment, the sample employed in Fig. 3C had been uploaded to maximise the prospect of detecting any binding. Thus the failure of p40ΔD3 to bind in this ELISA demonstrates that the D3 domain of p40 has a critical role in the heparin binding of IL-12.

### 3.4 Predictive docking of heparin oligosaccharides and IL-12

The predominant feature of a heparin binding site is a cluster of positively charged arginines and/or lysines, available for ionic interactions with sulfate and carboxyl groups of the highly anionic GAG chains [30]. As displayed in supplementary Fig. 1, the sequence of human p40 shows a single prominent cluster of 6 these basic residues within the 9 sequence positions 279–287 inclusive. We had proposed solely on the basis of sequence that the cluster of basic residues here was likely to constitute a heparin binding site [24]. Fig. S1 also shows that murine p40 possesses 8 basic residues in a longer but corresponding sequence, 269–282.

**Fig. 4:**
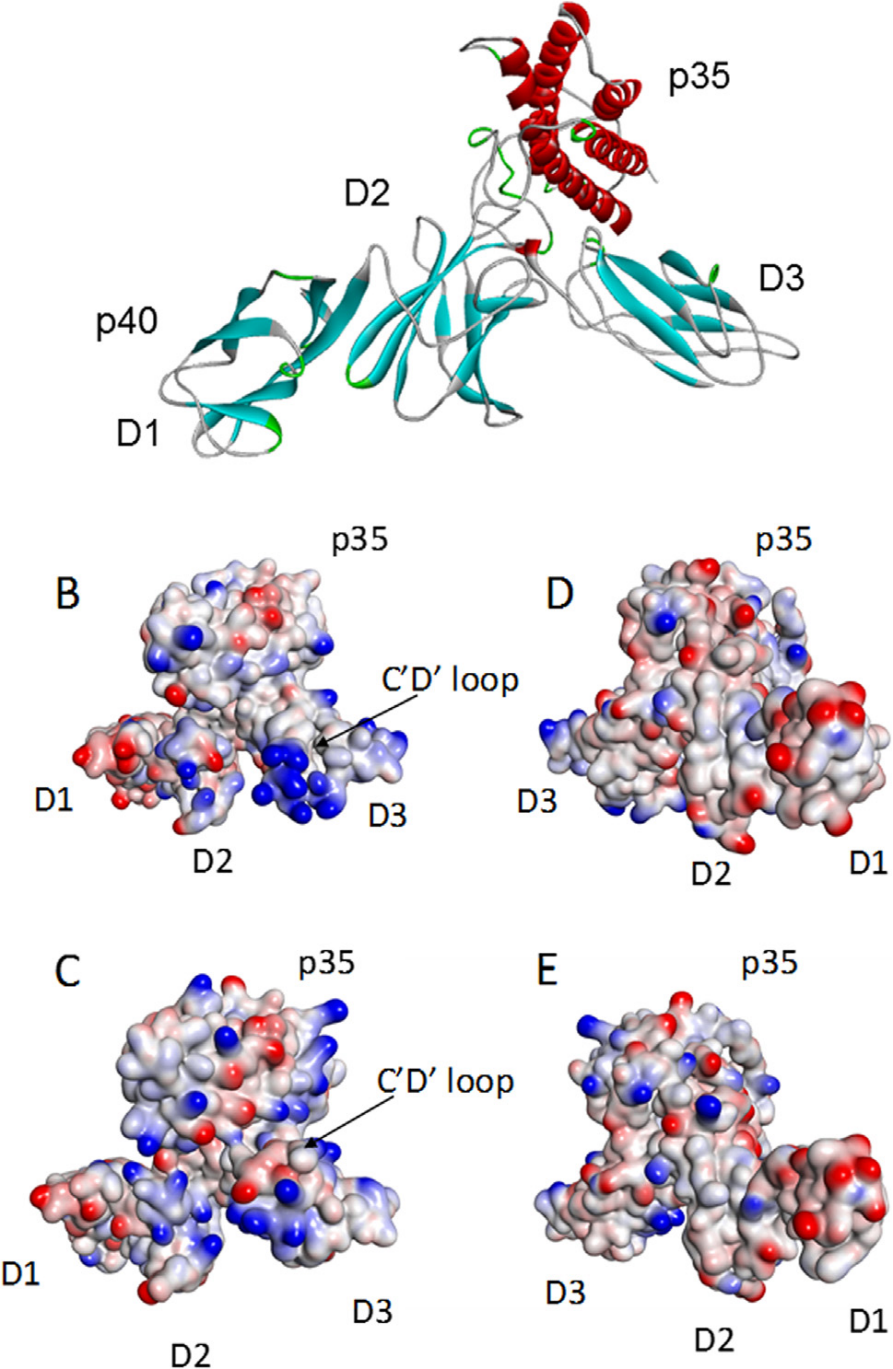
(A) A molecular model of the complete human IL-12 sequence based on the crystal structure 3HMX. The model is displayed as a ribbon, coloured according to secondary structure, with β-strands shown in aquamarine, and α-helices in red. (B–E) Molecular surfaces of human (B and D) and mouse (C and E) IL-12 coloured by interpolated charge (blue for basic, red for acidic). The D1 domains have a predominantly acidic surface, on which no heparin-binding site could be defined. Basic amino acid clusters are confined principally to domain D3, with contributions from D2 and from helix A of the p35 subunit. (D) and (E) show the reverse faces from those in (B) and (C). (For interpretation of the references to colour in this figure legend, the reader is referred to the web version of this article.)

In order to evaluate these basic residue clusters, together with other surface regions of p35 and p40, as possible locations of the heparin-binding sites on IL-12, we used systematic docking calculations that searched the entire protein surface, using heparin oligosaccharide model probes. We generated protein structures for human and murine IL-12 and their subunits using homology modelling as described in the Supplementary Material. A complete molecular model of human IL-12, with all loops completed by homology modelling, is shown in Fig. 4A. As shown by electrostatic surface maps, Fig. 4B–E, domain D1 of p40 has few basic surface residues in both the human and murine polypeptides (there being 81% identity between these two sequences). By contrast, as may be seen in Fig. 4B and C, in both species the surface of D3 has a large electropositive patch due to the basic sequences referred to above. On the reverse faces (Fig. 4D and E) there are fewer basic amino acids and these are dispersed amongst acidic and neutral residues.

**Fig. 5:**
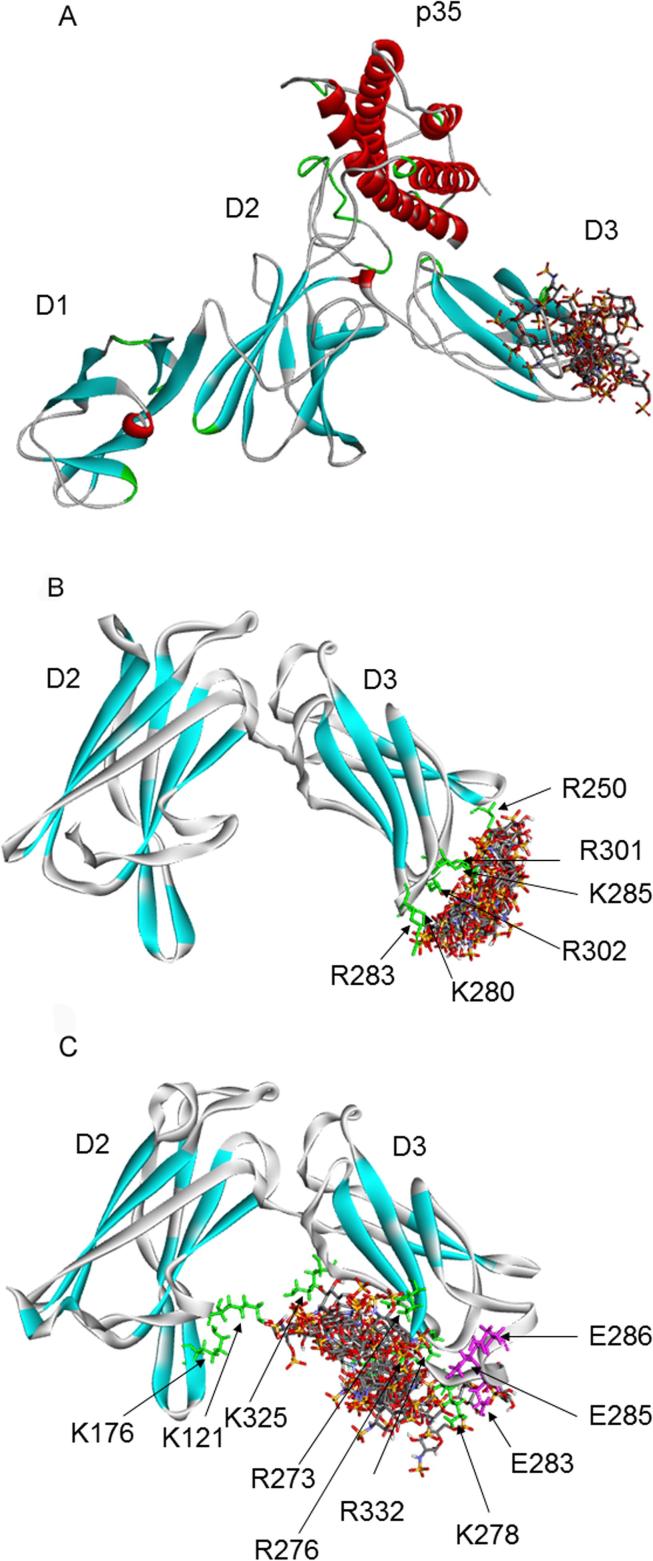
Predicted complexes of heparin oligosaccharides with IL-12 substructures. **(**A) The 3HMX model of whole human IL-12 docked with a heparin tetrasaccharide using Clus-Pro 2.0. (B and C**)** Heparin pentasaccharide docked with (B) human and (C) murine p40**:** in both cases only the D2 domains (left) and D3 domains (right) are shown. Amino acid residues predicted to be in contact with bound heparin are shown in green stick format and labelled. In all panels, polypeptide β-strands are shown in aquamarine, and red represents α-helices. The heparin oligosaccharides are shown in ball and stick representation with oxygen atoms in red and sulphur atoms in yellow. Side-chains of predicted heparin-binding basic residues are shown in green. In each panel the ten lowest energy orientations are overlaid. (For interpretation of the references to colour in this figure legend, the reader is referred to the web version of this article.)

**Supplementary figure 2:**
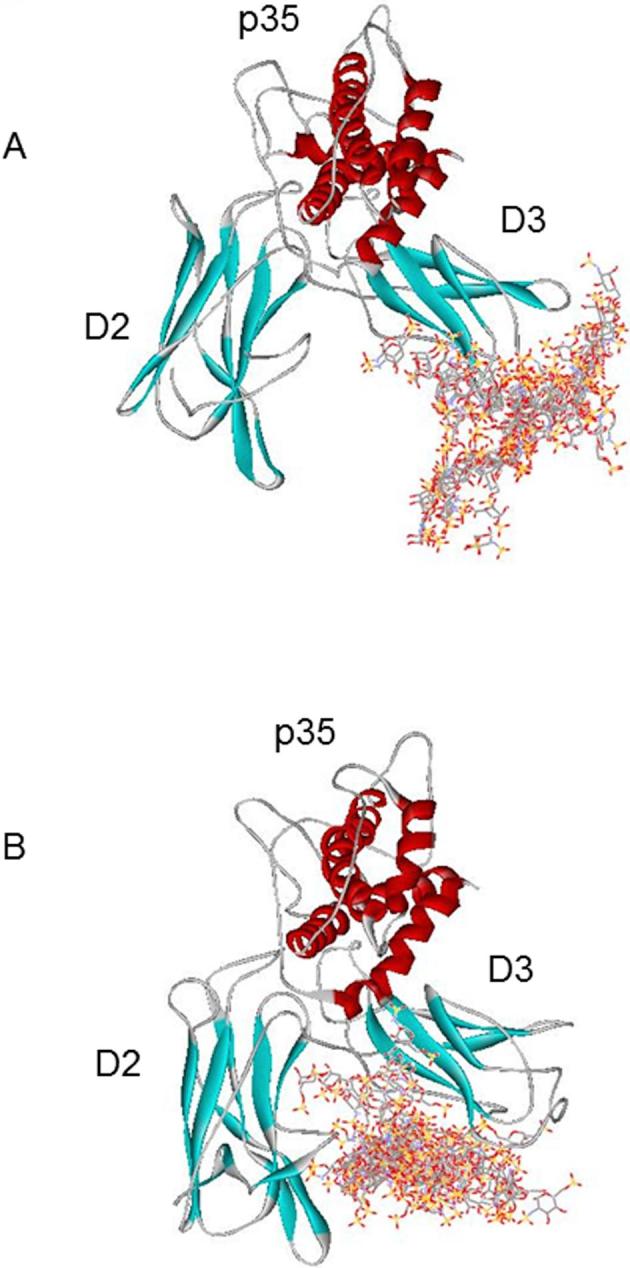


Docking calculations using Clus-Pro 2.0 and the 3HMX model of whole human IL-12 identified the electropositive patch in the D3 domain as the main feature of the heparin binding site (Fig. 5A). More detailed predictive docking calculations, using the programme Autodock 2.4, were performed for interactions of murine and human IL-12 subunits with heparin oligosaccharide ligands. Our protocol for this procedure has proved a reliable method for the identification of heparin binding sites on proteins, including those which, like IL-12, possess fibronectin-like domains [42]. Docking calculations did not identify any heparin binding site on the D1 domain of the p40 subunit from either species (data not shown). However, these calculations for both pentasaccharide and endecasaccharide heparin oligomers with models of the human IL12 p40 D2/D3 domains predicted the presence of a heparin-binding site formed from the three loops at the distal end of subunit D3 in p40, corresponding again to the basic patch in domain D3 (Fig. 5B, Fig. S2A). The human D3 domain is subject to C-α-mannosylation of tryptophan residue W319 in both recombinant and endogenously expressed IL-12 [43]. The same modification has been more recently observed in p40 of IL-23 [44]. This mannosyl residue is not represented in the available structures we have used in our modelling, but examination of the human p40 structure indicates that it is removed from our proposed heparin binding site we and therefore unlikely to affect this interaction.

Calculations for murine p40 predicted a heparin-binding site similar to that for the human protein (Fig. 5C). However, the more extended 282–292C′D′ loop at the distal end of D3 has a direct effect on the predicted heparin binding site in murine IL-12, both because of the increased size of the loop, and because the introduced sequence includes an acidic cluster of three glutamate residues (E283, E285, E286), which will engage in mutual charge repulsion with the highly acidic heparin oligosaccharides. Thus the heparin complex with murine D2/D3 (Fig. 5C) and with a D2/D3/p35 construct (Fig. S2B) shows heparin interacting with a line of basic residues running from the loops at the tip of D3 towards the interface with D2.

Docking calculations with the isolated human and mouse p35 subunits identified a potential heparin-binding site consisting of basic residues along helix A (data not shown), close to the interface between p35 and p40 subunits. As may be seen on the electrostatic potential-coded surfaces shown in Fig. 4B and C for human and murine IL-12 respectively, both models have a distinct basic patch including one face of D3, neighbouring basic residues in D2, and helix A of the p35 subunit, and it is possible that both the D2 domain and the p35 subunit contribute to binding of longer heparin polysaccharide molecules.

Interestingly, the docking calculations implicate the region of lowest homology between the human and murine p40 subunit sequences, the C′D′ loop of D3, as an element of the IL-12 heparin binding site. This loop shows the greatest variation between the species, with a considerable number of amino acid substitutions, 8 inserted residues in the mouse protein, and a different charge profile: the human sequence being more uniformly basic than the mouse (see Figs. S1, Fig. 4B and C). In addition this region is the least well defined structural element in the D3 domain, having a considerable degree of conformational freedom (see Supplementary Material). This precludes a precise static description of the heparin binding site from our docking calculations.

In summary, the predictive modelling results identify the D3 domain loops of the p40 subunit as the core heparin binding site for both human and mouse IL-12. Docking calculations using two distinct algorithms give results in close agreement. Calculations using longer heparin oligosaccharides raise the possibility that the D2 domain or the p35 subunit may contribute to an extended heparin binding site, particularly for mouse IL-12. All of the docking calculations identify non-contiguous, conformationally formed heparin binding sites involving several basic residues, and the inherent conformational flexibility of the loop region does not encourage the suggestion of a single defined pose for bound heparin.

## 4 Discussion

We previously determined that the immunostimulatory cytokine IL-12, both human and murine, binds to heparin and highly sulfated variants of HS [24]. This has since been confirmed independently by studies elsewhere which employed isothermal titration calorimetry to estimate the apparent dissociation constant for the binding of human IL-12 to heparin as being around 70 µM [45]. In the present study we examined further the heparin binding properties of human and murine IL-12. Firstly, we employed a panel of chemically modified heparins to investigate their heparin binding specificity profiles (Fig. 1). Each of the chemically modified heparin preparations chosen lacks sulfate at one of the three major sites of sulfation in the heparin disaccharide repeat unit: 2-*O*-sulfate, the 6-*O*-sulfate, or the *N*-sulfate. As the modified heparins are all able to interact with both human and/or mouse IL-12 to some extent, it can be concluded that none of the three positions of sulfation is absolutely required for binding to IL-12. Compared to other heparin binding proteins we have studied using the same chemically modified heparin preparations in this competitive ELISA approach, human IL-12 shows a distinct binding profile. For instance we established that the *N*-desulfated heparin is a largely ineffective competitor of the binding of the cytokine GDNF to immobilised heparin [34], whereas here with human IL-12 it proves to be an effective competitor (Fig. 1A). With human IL-12, re*-N*-acetylation of this preparation has little effect in this competitive activity. The human p40 subunit homodimer shows a very similar competitive binding profile to that of the human IL-12 heterodimer (Fig. 1B), indicating that the presence or absence of the p35 subunits had little or no effect on heparin binding. This provides evidence that the heparin binding site of IL-12 resides solely in the larger subunit. By contrast murine IL-12 shows a markedly different profile (Fig. 1C), as in this instance *N*-desulfated heparin is a relatively weak competitor which on re-*N*-acetylation becomes significantly more effective. Thus these data reveal a species difference between these two IL-12 heparin binding sites.

Next we investigated the ability of heparin to protect IL-12 from controlled proteolysis by the endoprotease Lys C (Fig. 2). We previously used this approach as part of establishing that the human cytokine betacellulin binds to heparin and HS [46]. For such work the endoprotease LysC is the enzyme of choice because it cleaves adjacent to lysine residues, which due to their strongly basic side chains and are along with arginines, key residues in heparin binding sites. In considering the outcomes here, it must it must be borne in mind that heparin might bind to a site on a polypeptide without necessarily offering such protection, and also that upon immunoblotting of the digestion products, only those fragments retaining major epitope(s) will be detectable. However, overall these experiments indicate that the p35 subunit shows only limited protection (Fig. 2A and C), and the p40 subunit is the primary locus of heparin protection. This further suggests that the p40 subunit is the location of the heparin binding site. A major effect of heparin on p40 digestion was that it prevented the appearance of large polypeptide fragments of slightly increased electrophoretic migration, indicating that one or more sites relatively near the p40 polypeptide chain terminii were being protected by heparin.

To further localise the heparin binding site of IL-12 we expressed a series of murine IL-12 constructs. These were psc70, a bioactive single chain construct encompassing both chains of the heterodimer [35], as well as p35, p40 and p40Δ3. Conditioned supernatants of all of these except p35 gave strong immunoreactive bands on immunoblotting, with migrations corresponding to the anticipated molecular weights (Fig. 3B). However despite the strong heparin-displaceable immunoreactivity demonstrated in the heparin binding ELISA for psc70 and intact p40, none was detectable for p40ΔD3 (Fig. 3C). Thus was despite the use of an elevated loading of the latter supernatant. This total loss of affinity for heparin in the truncated construct demonstrates that the heparin binding site resides in the carboxyterminal D3 domain of p40.

The aminoacid sequence of p40 contains a prominent basic residue cluster of 6 arginines and lysines in the case of the human protein, and 8 for the murine polypeptide. These lie in the C′D′ loop near the middle of the D3 domain, a region of low sequence homology between the human and murine proteins due to an 8 residue insertion in the mouse sequence. In both species this sequence cluster forms the core of a large exposed electropositive patch on the concave face of the D3 domain. Through the use of well-established molecular docking simulations we show that binding of heparin oligosaccharides to these sites is entirely feasible, although the detailed interactions differ between the human and murine IL-12s due to structure divergence in this region. In the molecular structures available for the p40 subunit, the C′D′ loop, along with the aminoterminal sequence, are the two largest unresolved portions of the polypeptide. This implies conformational flexibility of the C’D’ loop in both species.

The heparin binding sites in human and murine p40s we presently propose involve the prominent clusters of basic residues that, on the basis of sequence alone, we had previously suggested might be important in this interaction [24]. More recently, Jayanthi et al [45] independently proposed two possible heparin binding sequences within human p40, the longer of which is essentially the C′D′ loop of D3 and in agreement with our proposal. Their shorter sequence, Leu117-Lys127, is exposed on the surface of D2. Our present predictive modelling studies of heparin-human IL-12 interactions identify the former sequence, with no involvement of the latter for the relatively short heparin oligosaccharides modelled. By contrast, our modelling of the murine binding site does show some involvement of this D2 sequence together with other basic residues from a neighbouring β-strand.

In a number of studies the contribution of individual amino acid residues within heparin binding sites has been probed by site-directed mutagenesis, although precise delineation of the key residues involved is often difficult. Our recent mapping of the extended heparin binding site on the BMP antagonist gremlin [47] has shown that the substitution of as many as six basic residues was not able to abrogate heparin binding completely. By contrast to gremlin, in which the conformation of the polypeptide within the heparin binding site is constrained by intra-chain disulphide bridges of the TGF-β fold, our predictions for IL-12 implicate residues within the unordered and relatively unconstrained loops joining the packed α-helices of D3. Because of the probable conformational flexibility of these loops it is likely that the substitution of any residue within them, irrespective of sidechain charge, will alter sidechain packing and therefore the conformation of the loop within the mutant variant. Any such conformational change might well affect affinity for heparin, even when if the targeted residue has no direct interaction with the polysaccharide. Thus probing these loops by mutagenesis is unlikely to reliably identify the key contact residues.

The human p40 D3 domain contains tryptophan W319, which as referred to above is the site of mannosylation. This residue also lies at the start of WSXW motif, which in most thrombospondin repeat type 1 domains, and some fibronectin type III domains, forms the base of a so-called tryptophan ladder involving basic residues on neighbouring β-strands (for review see Olsen and Kragelund [48]). There is some evidence that one function of such ladders is to associate with anionic components of the extracellular matrix, including heparin and related polysaccharides. Within human D3, there is a truncated tryptophan ladder involving only the tryptophan tetrapeptide motif and a single basic residue R309, as shown in Fig. S4. Our unbiased binding protocols found no potential interaction with this tryptophan step. In any case there is no tryptophan step in murine p40.

A detailed structural elucidation of the interaction of IL-12 with its high-affinity receptor polypeptides β1 and β2 is not yet available. However, Yoon et al. have proposed that the exposed face of p35 is important in IL-12/IL-12 receptor interactions [21]. Moreover, the binding site for the Mab ustekinumab, which neutralises IL-12 activity, is located on the exposed long axis of the D1 domain of p40 [22], well removed from the heparin binding sites we propose here. Even in the hexameric IL-12/IL-12 receptor signalling complex postulated by Yoon et al. [21], the heparin binding sites we propose remain exposed. Very recently Bloch et al. [23] have produced a high resolution structure of the related IL-23 complexed with the extracellular domain of IL-23R. This shows that the major contact is between D1 of this receptor chain and the peptide loops of an exposed end of p19, although there is also minor contact with D1-D2 hinge region of p40, inducing a 5–10° flexing of the D1-D3 orientation. The surface of D3 remains exposed in this complex.

This apparent separation between the likely receptor binding sites and the heparin binding sites is consistent with our observations (data unpublished) that addition of sulfated GAGs including heparin and HS neither inhibits nor potentiates murine IL-12 bioactivity in the KY-1 cellular assay [33]. Subsequently, in studies of human IL-12 in human natural killer cell line NK-92MI and with human peripheral blood mononuclear cells, heparin was found to potentiate IL-12 activity [49]. Although this apparent species difference may arise from variations between the assay systems employed, it is also consistent with the difference we propose between the heparin binding sites of murine and human IL-12.

Irrespective of the effect of heparin and HS on IL-12 activity, it is likely that a major physiological effect of this interaction is the tethering of the cytokine close to its sites of secretion within the tissues by binding to HS chains of adjacent cell surfaces and extracellular matrix [30]. Indeed Jayanthi et al. [49] have reported the binding of rhIL-12 to cell surfaces which was markedly increased by the addition of soluble heparin, which may be indicative of heparin/HS providing a reservoir of IL-12 at the cell surface HS. This binding would function to localise the immunoregulatory activity of IL-12. Such localised, paracrine activity of IL-12 has been proposed to underlie the effectiveness of IL-12 secreting chimeric antigen receptor (CAR)-modified T cells in eliminating experimentally induce tumours [50]. Since the cytokines IL-12 and γ-IFN constitute a positive feedback loop for the stimulation of T_H_1 immune responses, and the latter is also a heparin/HS binding cytokine [31], this suggests an important role for HS in the paracrine stimulation of cellular immune responses. As the p40 subunit is also a component of the immunostimulatory cytokine IL-23, it is a reasonable speculation that it too will be localised in this way.

In summary, it is clear that both murine and human IL-12 bind to heparin and related polysaccharides via the carboxyterminal D3 domain of their p40 subunits. The conformational plasticity of the exposed loops in this domain render precise delineation of the binding site impractical, but our molecular simulations predict the C′D′ loop of D3 as being central to this interaction. This loop contains a prominent cluster of exposed lysine residues and being near the C-terminus of the polypeptide chain, is consistent with the observed protection by heparin from clipping with the endoprotease Lys-C. This loop is the region of lowest homology between the two orthologs, showing differences in sequence, length and charge. It would therefore appear that the requirement to interact with the sulfated polysaccharide ligand has not constrained the polypeptide from undergoing considerable evolutionary divergence within this loop. Such divergence of the heparin binding site of a protein between mammalian species is somewhat unexpected. Indeed, in a comparative study of FGF-FGFR-heparin interactions, in which the structure of human FGF1-FGFR2-heparin complex was compared to models of the *Drosophila melanogaster* FGF-FGFR2-heparin and *Caenorhabditis elegans* egl17-egl15-heparin complexes*,* it was deduced that despite numerous amino acid substitutions involving key residues, the heparin binding site was evolutionarily well conserved in terms of the positioning of the bound heparin chain [51]. By contrast, within the BMP-2/BMP-4 subfamily of the bone morphogenetic proteins, these two cytokines bind heparin via aminoterminal sequences which show both sequence divergence and conformational flexibility (for review see [52]). It is also notable in this context that osteopontin is a heparin binding protein which is intrinsically unstructured with considerable conformationally flexibility in solution [53]. Our present predictions with IL-12 add further insight into the evolution of heparin-protein interactions. In this case we conclude that as long as evolutionary changes do not actually abolish heparin binding, considerable flexibility in the structure of heparin binding sites is permissible. This implies that the functional importance of heparin binding does not necessarily depend on a precise orientation and alignment of the bound polysaccharide chain with respect to the domains of the polypeptide.

## Declaration of interests

None.

## Figures and Tables

**Supplementary figure 3:**
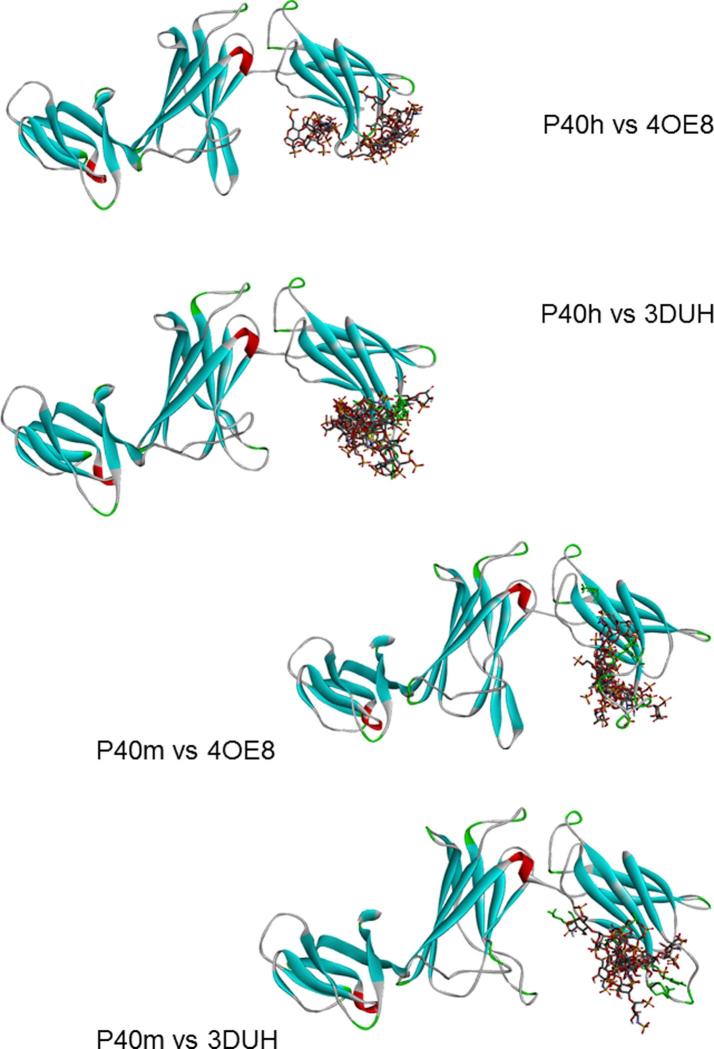


**Supplementary figure 4:**