# Supplementary material for: The localisation of the heparin binding sites of human and murine interleukin-12 within the carboxyterminal domain of the P40 subunit
Source: Cytokine. 2018 Oct;110:159–68. doi: 10.1016/j.cyto.2018.04.014 (PMC6109204; doi:10.1016/j.cyto.2018.04.014)
Supplement: Supplementary data 1 [file mmc1.docx]

**Supplementary Material**

Garnier et al.

Homology modelling was performed using Modeler [S1] running as a module in the Insight software (Accelrys, Cambridge, UK) or using the Swiss-Model server (http://swissmodel.expasy.org/) in its 2014 implementation [S2]. Crystal structures of human IL-12 were obtained from the RCSB Protein Data Bank; 1F45 [S3] and 3HMX (human IL-12 complexed with ustekinemab [S4]) for both p35 and p40 subunits. For the p40 subunit only, crystal structures 1F42 [S3], 3DUH [S5] and 4OE8 [S6] were used. Mouse IL-12 and its subunits were modelled using the murine p40 sequence NP_001152896 and the p35 sequence NP_001290173.1 on the basis of the crystal structures 1F45, 1F42 and 3HMX.

Generation of molecular models for IL12 and its subunits, and docking calculations with heparin ligands, took place in two stages. The Modeler programme was used for homology modelling on the basis of crystal structures 1F42 and 1F45, with Autodock 2.4 in the docking calculations for detailed analysis of individual and paired subunits of IL-12. The more recent crystal structures 3HMX, with p40 subunits from 3DUH and 4OE8 were modelled using the Swiss-Model server in its most recent (2014) version [S2] The Clus-Pro server v 2.0, which offers an option to use a heparin tetrasaccharide ligand [S3], was used for some docking calculations.

Some crystal structures of human p40 (1F42, 1F45, 3DUH) have a few missing residues corresponding to short exposed loops in the D1 and D2 domains; these are resolved in 3HMX and 4OE8 (see Fig. S1). More striking is that all structures other than 3DUH show gaps in the polypeptide chain between residues 279 and 288, which corresponds to the loop between the β-strands C’ and D’ in D3 (Fig. S2A). In addition, 1F45 and 3HMX have missing residues in the E’F’ loop of D3. Homology modelling was used to ‘mend’ these loops using the full length sequence of human IL-12 p40 (AAD56386) aligned with the crystal structures. In the p35 subunit, missing residues were also replaced by homology modelling using the human sequence NP-000873.2.

There is no experimental high resolution structure of murine p40, but the human and murine sequences show a 60% level of amino acid conservation. However, there is one region of low homology, including 8 inserted residues, in the murine sequence towards the carboxy terminus: this corresponds to the unresolved C’D’ loop of D3 in the crystal structures of human IL-12. A homology model of murine IL-12 was generated which closely followed the backbone of the human structures 1F42 and 1F45 (not shown). Residues 160-164 and 282-292 in the mouse sequence, (see Fig. S1) were modelled as loops. The shorter loop, 160-164, had little conformational freedom. For the longer C’D’ loop, which includes the 8 residue insertion, ten conformations were generated using the Modeler programme, from which four were selected as representing the scope of conformational flexibility in this region. These different loop conformations of mouse IL-12 did not differ in the geometries of their predicted complexes with heparin (not shown).

Confirmatory docking calculations using the Clus-Pro 2.0 protocol were applied to p40 models based on the IL-23 crystal structures 4OE8 and 3DUH; these structures are of good resolution (1.74 and 2.30 Å respectively) and 3DUH has an intact C’D’ loop in D3. In each structure, the binding site was located primarily at the tips of the loops for the human p40 model and on the concave face, towards the D2 unit, for the mouse p40 model (Fig. S3).

In some extracellular protein domains a so-called Trp ladder is found, characterised by a WSXW consensus motif together with aligned Trp and Arg residues [S7]. In some instances the resulting linear array of multiple basic Arg residues can form the basis for a heparin binding site [S8]. In the case of the IL-12 p40 D3 domain, a WSXW motif is present and a single neighbouring Arg gives rise to a truncated ‘single-step’ Trp ladder (Fig. S4). The heparin binding site is not predicted by our docking protocol to include this feature. Murine p40 lacks the WSXW motif.

**References**

[S1] A. Šali, and T.L. Blundell. Comparative protein modelling by satisfaction of spatial restraints. J. Mol. Biol. 234 (1993) 779-815.

[S2] M. Biasini, S. Bienert, A. Waterhouse, K. Arnold, G. Studer, et al. SWISS-MODEL: modelling protein tertiary and quaternary structure using evolutionary information.  (2014). Nucleic Acids Res. 42 (2014) W252-W258.

[S3] C. Yoon, S.C. Johnston, J. Tang, M. Stahl, J.F. Tobin, and W.S. Somers. Charged residues dominate a unique interlocking topography in the heterodimeric cytokine interleukin-12. EMBO J. 19 (2000) 3530-3541.

[S4] J. Luo, S.-J. Wu, E.R. Lacy, Y. Orlovsky, A. Baker, A. et al. Structural basis for the dual recognition of IL-12 and IL-23 by ustekinumab. J. Mol. Biol. 402 (2010) 797-812.

[S5] P.J. Lupardus and K.C. Garcia. The structure of interleukin-23 reveals the molecular basis of p40 subunit sharing with interleukin-12. J. Mol. Biol. 382 (2008) 931-941.

[S6] J. Desmet, K. Verstraete, Y. Bloch, E. Lorent, Y. Wen, et al. Structural basis of IL-23 antagonism by an Alphabody protein scaffold. Nat. Commun. 5 (2014) 5237. doi: 10.1038/ncomms6237.

[S7] J. G. Olsen and B.B. Kragelund. Who climbs the tryptophan ladder? On the structure and function of the WSXWS motif in cytokine receptors and thrombospondin repeats. Cytokine Growth Factor Rev. 25 (2014) 337-341.

[S8] H. Tossavainen, T. Pihlajamaa, T.K. Huttunen, E. Raulo, H. Rauvala, et al. Permi P,

The layered fold of the TSR domain of P. falciparum TRAP contains a heparin binding site.

Protein Sci. 15 (2006) 1760-1768.

**Figure Legends**

Figure S1. Alignment of the aminoacid sequences of human and murine p40. The human sequence, NCBI Entrez Protein accession number AAD56386, is represented by the one letter code. The basic residues in the human sequence identified in this study as contacts with the docked heparin oligosaccharides are shown in bold italics. For the murine sequence (AAA39296), identical residues are represented by a dot, and substituted residues are indicated by letter. Sequence gaps to optimise alignment are shown by a dash. The underlined residues in the murine sequence show the major basic sequence cluster. The horizontal lines above the human sequence shows the extent of residues resolved in the crystallographic structures 1F42, 1F45, 3HMX, 4EO8 and 3DUH. The starts of the D2 and D3 domains are indicated by vertical dashed lines labelled in italics.

Figure S2: Docked complexes of heparin endecasaccharide, with (A) human, and (B) mouse D2/D3/p35 substructures. Peptide chain shown in ribbon format, α-helices in red and β-strands in aquamarine; ten lowest energy orientations of the heparin endecasaccharides shown in stick format.

Figure S3: Docked complexes of a heparin tetrasaccharide with human and murine p40 models based on the crystal structures 4OE8 and 3DUH. All three p40 domains are shown with the D3 domain on the RHS; α-helices in red and β-strands in aquamarine. The heparin tetrasaccharide is shown in ball and stick representation with oxygen atoms in red and sulphur atoms in yellow. The ten lowest energy orientations are overlaid.

Figure S4: The single-step tryptophan ladder in human p40. Human IL-12 domains D2 and D3 (in ribbon representation coloured by secondary structure; β-sheet coloured blue), with docked heparin endecasaccharides (in stick representation, coloured by element: carbon atoms grey, oxygen red, nitrogen blue and sulphur yellow). The sequence W319 S320 E321 W322, on the C-terminal β-strand, together with Arg 309 on the adjacent strand, forms a single-step Trp ladder. The two Trp residues are shown in ball and stick representation, with Arg 309 in space-filling representation.
