# Supplementary figures and images for: The localisation of the heparin binding sites of human and murine interleukin-12 within the carboxyterminal domain of the P40 subunit
Source: Cytokine. 2018 Oct;110:159–68. doi: 10.1016/j.cyto.2018.04.014 (PMC6109204; doi:10.1016/j.cyto.2018.04.014)

Fig. S1

Garnier et al

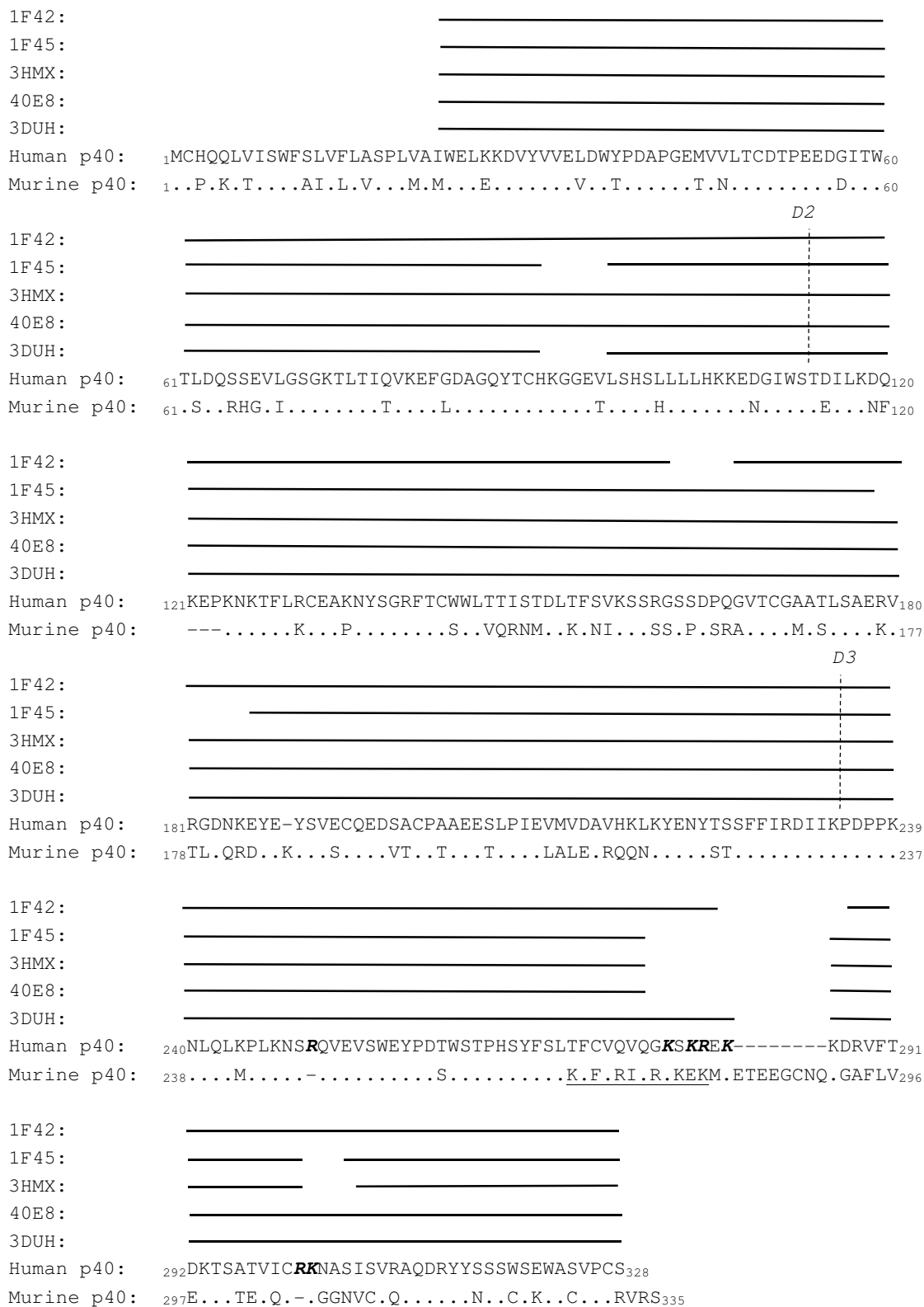

Supplement: Supplementary data 2 [file mmc2.pdf]
